# Strengthening the Acute Flaccid Paralysis (AFP) Surveillance Component of the Polio Eradication Initiative through Short Message Service (SMS) Reminders; Experience from Sokoto State, Nigeria 2014

**Published:** 2018-07-03

**Authors:** Oluwasegun Joel Adegoke, Marina Takane, Oladayo Biya, Martin Ota, Bolatito Murele, Frank Mahoney, Patrick Nguku, Hiromasa Okayasu

**Affiliations:** 1 African Field Epidemiology Network, Nigeria Country Office, Abuja, Nigeria; 2 World Health Organization, Geneva, Switzerland; 3 World Health Organization Country Office, Abuja, Nigeria; 4 Centers for Disease Control and Prevention, Atlanta, USA; 5 World Health Organization -Regional Office for Africa, Brazzaville, Republic of Congo

**Keywords:** Poliomyelitis, Acute Flaccid Paralysis, KAP, Healthcare worker

## Abstract

Eradication of poliomyelitis remains a public health priority due to the paralytic effects of the virus on children and impact on global health system. However, existing gaps in surveillance can hinder eradication. Improved timeliness of identification and reporting of acute flaccid paralysis (AFP) cases with further confirmation of Wild Poliovirus (WPV) in stool samples, can help Nigeria achieve the performance indicators of non-polio AFP rate of ≥ 2/100,000 population aged < 15 years and ≥80% stool sample collection adequacy.

To ascertain the awareness of AFP case definition and detection by health care workers and to evaluate the impact of SMS-based reporting on the AFP surveillance system the study was conducted from November 2013 to July 2014.

In Sokoto state, 112 health facilities (focal sites) were operational and participated in this study. All AFP focal points for the 112 facilities were included in the study. In addition to AFP focal points, two clinicians per facility where possible, were included in the study. The study focused exclusively on reports from focal sites. The methodology was a one group pretest-posttest design conducted in 3 phases. 1) Pre-intervention Knowledge, Attitude and Practices (KAP) survey, 2) SMS implementation and 3) Post-intervention KAP. Results were analysed using the independent sample t-test to assess the increase in knowledge, attitudes, or practice scores pre- and post- training.

The study showed improved knowledge gap of health care workers on AFP surveillance between pre and post intervention. It shows that this approach of improved surveillance will be effective in countries in hard to reach, access compromised or countries/place without sufficient surveillance staff.

## Introduction

Poliomyelitis remains endemic in three countries – Afghanistan, Pakistan, and Nigeria. Nigeria appeared to have interrupted wild poliovirus (WPV) transmission after the WPV case in July 2014. However, in July 2016, continued WPV circulation was detected in Borno, a northern state with ongoing conflict^1^. Polio cases are detected through surveillance for acute flaccid paralysis (AFP) which entails rigorous, timely AFP identification and reporting, and confirmation of WPV in stool samples^2^. Identification of AFP caused by polio is significant because, for every child affected by paralytic poliomyelitis, approximately 200-1,000 children may be infected with WPV without paralysis. Therefore, failure to detect cases early enough and to respond promptly through supplementary immunization activities (SIA) will hinder the ability to eradicate polio. Consequently, sensitive surveillance is required to monitor the epidemiological situation as an indicator of programme impact, and to guide the immunization strategy, as a means to achieve and sustain eradication. The two major AFP surveillance performance indicators are 1) non-polio AFP rate of ≥ 2/100,000 population aged < 15 years old and 2) ≥80% stool sample collection adequacy. In addition, the Global Polio Eradication Initiative (GPEI) utilizes environmental surveillance, in which sewage samples are collected and analysed to provide supplementary information, particularly in urban populations where AFP surveillance is absent or questionable, persistent virus circulation is suspected, or there is a high risk of virus re-introduction. In Nigeria, environmental samples are currently collected at 43 sites in 10 states and the Federal Capital Territory^2^.

In 2015, Nigeria achieved a NP-AFP rate of 17.1/100,000 and 98% stool sample adequacy respectively^2^. However, the recent discovery of undetected transmission in Borno highlighted the challenge of maintaining the quality of surveillance in inaccessible areas^1^. Furthermore, a community study indicated that awareness and knowledge of AFP surveillance is low among the community in Northern Nigeria despite the polio eradication campaign that has been ongoing for more than a decade^3^. These suggest the existence of gaps in AFP surveillance, which need to be closed to achieve polio eradication.

In Nigeria, a focal point for AFP surveillance is assigned at each AFP reporting site, responsible to immediately report any suspected AFP cases to the Disease Surveillance Notification Officer (DSNO) at the district level and to conduct active AFP case search. The DSNO examines each case identifying children with symptoms of paralysis and collects adequate amounts of stool samples from the child within a specific time period. The DSNO is responsible for transporting the sample (via reverse cold chain) to a certified laboratory for confirmation and genetic classification of any isolated virus into non-polio, Sabin or WPV^4^. The DSNO is also responsible for conducting regular “active surveillance” visits to health facilities to remind health workers and focal points to report AFP cases immediately, although in practice this does not always happen regularly because of logistical challenges and other factors. The active case search activities by the focal point and by the DSNO are important as some clinicians who see AFP cases in their everyday practice are either not aware of the reporting system or forget to report unless reminded regularly.

The increased use of mobile phones and expanded network coverage over the last decade has provided a unique opportunity for surveillance using phone. Mobile phone subscription in sub-Saharan Africa increased annually by 49% between 2002 and 2007 as compared to a 17% increase in Europe^5^. According to the Nigerian Communication Commission (NCC), in September 2015 Nigeria had approximately 150 million active subscribers, 98% of which are GSM mobile lines^6^. Initiatives using short message service (SMS) to collect data already exists in Nigeria and other parts of Africa especially in health care service delivery through reminders and feedback. The application of the technology for AFP surveillance poses an opportunity for real-time data collection for quick action and suitable feedback mechanism to health care workers.

We conducted a pilot study in the state of Sokoto to evaluate the impact of refresher training and weekly SMS-based reminders and reporting on the AFP surveillance system. The specific objectives are to 1) assess the changes in Knowledge, Attitude and Practice (KAP) among surveillance focal points and clinicians and 2) assess the progress in improving AFP surveillance indicators (e.g. reporting rate, timing).

## Methods

### Study site and population

The study was conducted in Sokoto state, in Northwest Nigeria. There are two major ethnic groups in the state: Hausa and Fulani. The state is divided into 23 local government areas, has 675 health facilities with 127 of them listed as AFP reporting centres, which are categorized as high, medium or low AFP reporting sites. In Sokoto State, 112 of these sites were operational and participated in this study. All AFP focal points for the 112 facilities were included in the study. In addition to AFP focal points, two clinicians were selected per facility where possible for inclusion in the study.

### Study design

The experimental study has a one-group pretest-posttest design. It was conducted in three stages. The first stage was the baseline assessment of the knowledge, attitude, and practice (KAP) of the health care workers. The second stage entailed two interventions, the first part being the training of both focal points and clinicians and the second part being the use of SMS reminders for AFP reporting for a period of 36 weeks in addition to the routine method. The third stage of the study was the follow-up assessment of health care worker awareness and knowledge of AFP and the surveillance process.

### Baseline assessment of AFP detection and notification of health care workers

The baseline KAP of AFP focal points and clinicians from the health facilities was evaluated using a questionnaire, which assessed knowledge of signs of AFP, causes, samples and timing, reverse cold chain and reporting channel of AFP when detected (Table 1). The health care workers were then given a short training course on AFP detection and the surveillance system using standardized training materials. After that the participating health care workers (focal points, clinicians, and DSNOs) were trained on the processes involved in the SMS reminders and reporting system, and their contact mobile telephone numbers were recorded. They were requested to continue the traditional AFP reporting system in addition to the SMS method.

### Use of SMS reporting system

The SMS reporting method is a semi-automated system using the RapidSMS platform that automatically sends out reminders to health care workers at a designated time, receives reports and aggregates the information on the system dashboard. RapidSMS is an open source SMS application platform written in the Python programming language^7^. During the implementation phase of the SMS-based reminder system, SMS reminders were sent to the focal points and clinicians every Friday with the following messages tailored according to the profile of the health worker

Clinicians: “Please report any suspected case of AFP in your health facility to the AFP focal point.”

AFP focal points: “Do you have any new case of AFP? Please remember to report immediately any acute flaccid paralysis cases immediately to the DSNO for investigation. Ask all health workers in the facility about any AFP cases this week that they haven’t reported.”

DSNO: “Please report all AFP cases for the week.”.

The focal point was reminded to do active case search and report all suspected AFP case to the DSNO for investigation. A second reminder was sent on the following Monday to all participants who had not yet responded.

Each week reports from the FPs and DSNOs were checked and discrepancies resolved immediately. Even if no AFP cases were seen at the facility that week, the FP is required to report zero cases to the DSNO (zero reporting).

Once a suspected AFP case was reported by SMS, an automated SMS reply was sent to the focal point requesting the case information (e.g., name, sex, date of onset, date reported). The system was managed by a data clerk who sorted the health care worker reports categorizing them into ‘zero reporting’ and ‘suspected AFP ‘using the system tool. For suspected AFP cases, the data clerk sorted feedback based on reported information that included the name, age, date reported, the number of AFP cases reported at the site, and the number of AFP cases reported to the DSNO.

### Post-training assessment of KAP of health care workers and review of clinic registries

After sending SMS reminders to all focal points for 36 weeks, the investigators visited the same facilities and administered the same standardized KAP questionnaire as in the pre-intervention phase. During the follow-up assessment, active case search through register review was conducted: potential AFP cases in facility records of relevant hospital departments (e.g., outpatient, paediatric, physiotherapy, neurology, etc.) were sought for missed AFP cases using standardized criteria. The interviewer looked for AFP relevant symptoms (e.g., paralysis, unsteady gait, pain in legs, inability to walk and weakness) in the diagnosis registers and crosschecked with the AFP line list for the preceding 3 months (May 1, 2014, to July 31, 2014).

### Data collection

Data collectors were selected from among the Nigeria Field Epidemiology Laboratory and Training Program (NFELTP) students for the pre and post evaluation. They received training on AFP case definitions, study protocol, and data collection framework for the KAP survey. A field pilot was also conducted successfully in two health facilities before commencing on the main study. An active case search data through register review questionnaire was obtained post follow-up assessment. For the implementation phase, data was collected using the AFP SMS reporting system.

### Data analysis

All KAP data were cleaned and entered into an electronic database using Epi-info 7 software (US Centers for Disease Control and Prevention) and analysed using SPSS (IBM Statistical Package for the Social Sciences, Version 21). We determined the proportion of health care workers that participated in the baseline and follow-up KAP assessment. Using an independent sample t-test (2-tailed), we assessed the increase in knowledge, attitudes, or practice scores pre- and post- training. An independent sample t-test was used because the pool of respondents pre- and post-intervention changed considerably. There were four questions selected for knowledge, 2 for practices and 2 for attitudes. The mean score was calculated by adding up the score (Yes=1, No=0) on correct responses for questions to determine KAP. Scores range from 0 – 8 (Table 1).

Data from the weekly SMS reporting system were analyzed for reporting rate. Reporting sites were categorized into “Every week”, “Variable reporting” and “Silent reporting”.

### Ethical review

Ethical clearance was obtained from the Sokoto Primary Health Care Development Agency Board, Sokoto State, Nigeria.

## Results

### Baseline assessment

A total of 223 clinicians from 112 health facilities participated in the baseline KAP assessment. During the intervention, all participating health facilities were operational in active surveillance. One hundred and six (47.5%) of these clinicians were AFP focal points of various health facilities. The majority of the clinicians and AFP focal points were community health extension workers (CHEW). During the baseline survey, 80% of focal points answered questions correctly on AFP case definition, whereas only 48.9% of clinicians knew the proper case definition for AFP. Even fewer clinicians (19.7%) were found to have knowledge of active case search (Table 2).

### Use of SMS reporting system

**Figure 1:**
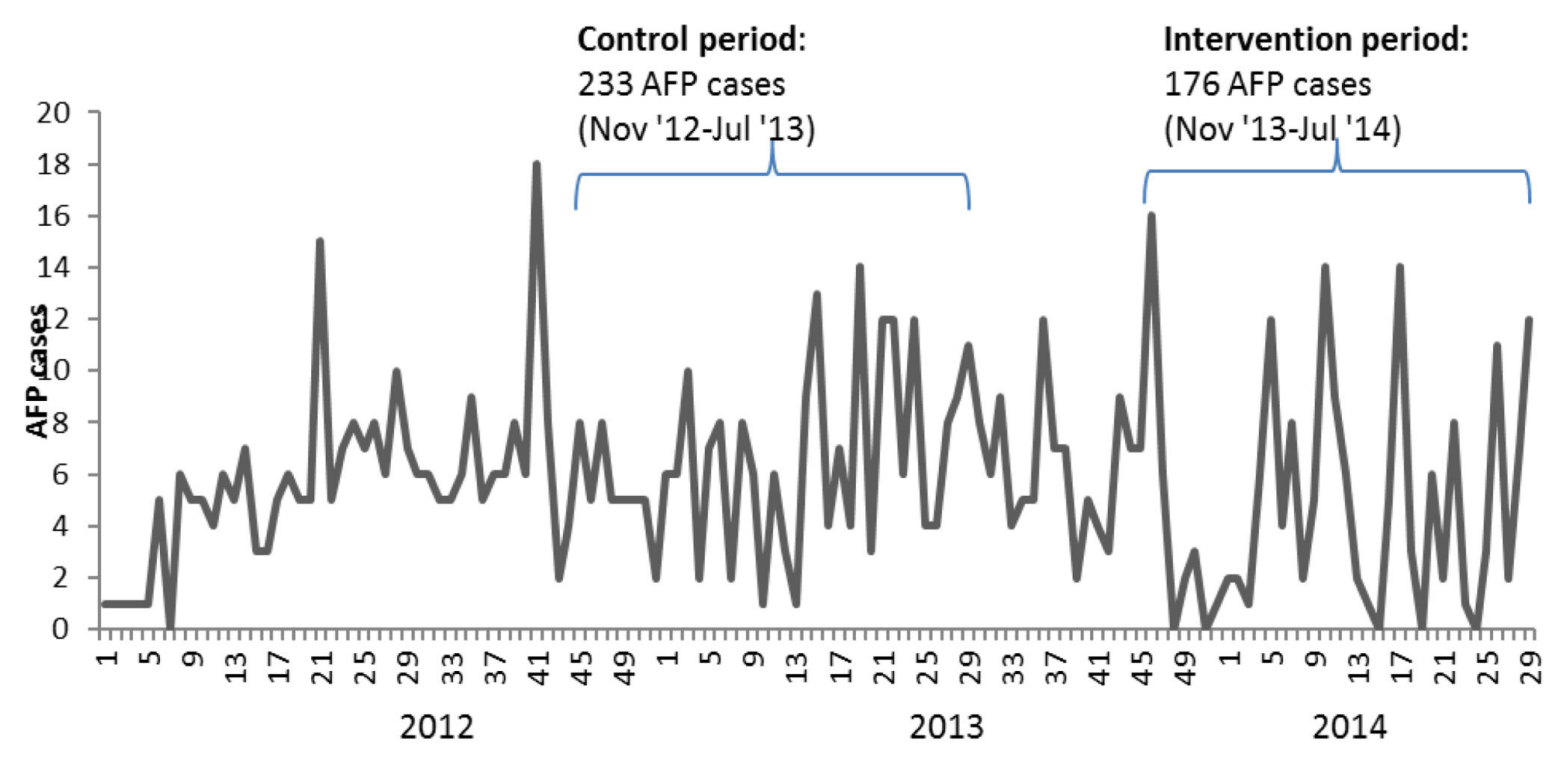
Number of AFP cases reported in Sokoto by week, 2012-2014

**Figure 2:**
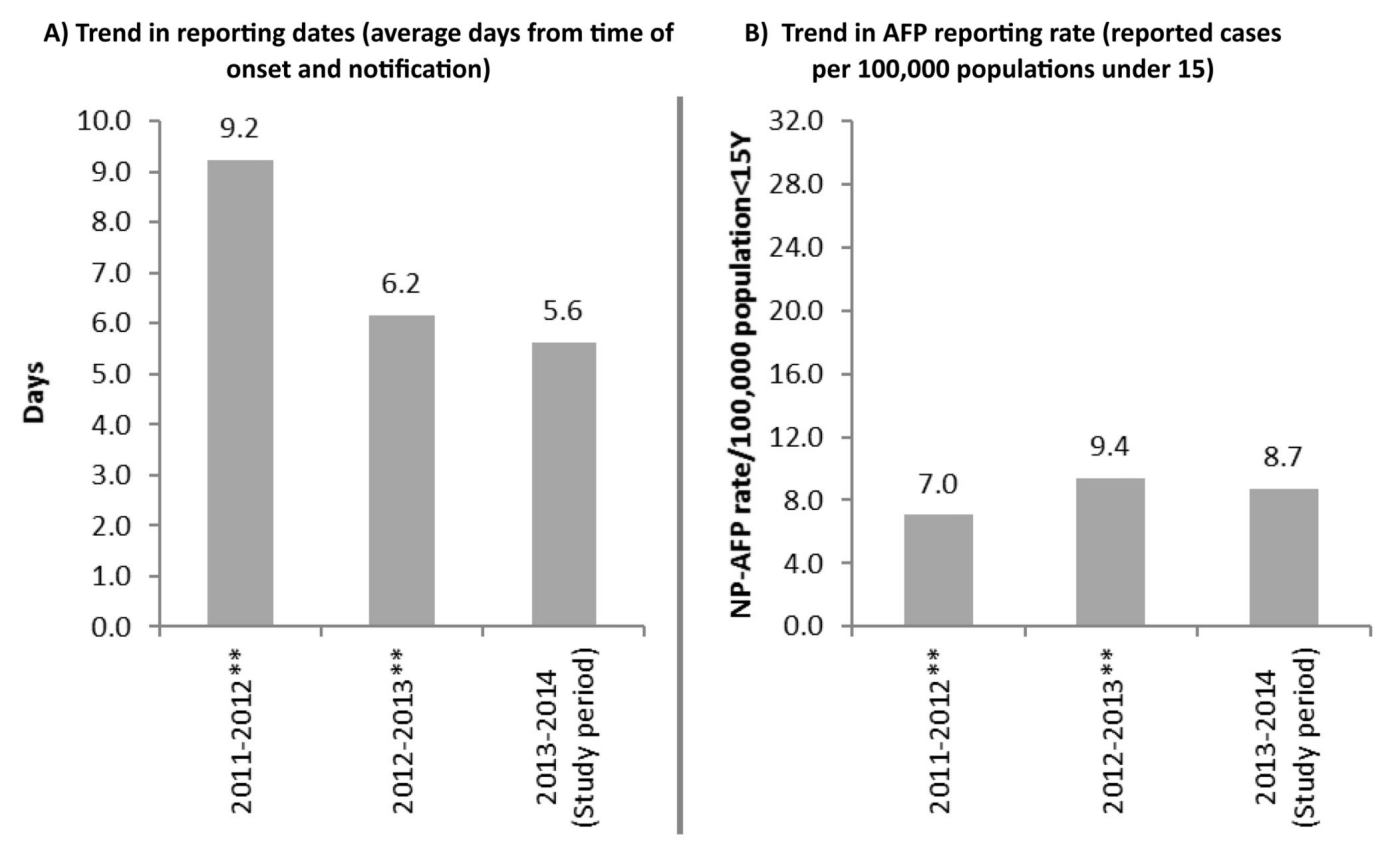
SMS impact on reporting rate and time (November to June in 2011-12, 2012-13 and 2013-14).

The SMS reminder intervention system was operational for 36 weeks. The intervention period of November 2013 – July 2014 had an average of 54% completeness of report from AFP surveillance focal points. During the intervention period, a total of 176 AFP cases were reported through the SMS reporting system as compared to 233 AFP cases reporting through the official reporting channel for the same reporting period in 2012/2013 (Figure 1). Eighteen percent of confirmed AFP cases were reported through the SMS reporting system for the reporting period. The SMS reporting system picked up only one case not reported through the official reporting channel. Review of registers in surveillance sites did not find any missed AFP case during the SMS-based intervention. The average number of days between the date of AFP onset and that of notification during the study period was 5.6 days as compared to 6.2 and 9.2 in the similar period one and two years previously. NP-AFP rate per 100,000 population < 15Y was also comparable to similar periods in the previous years (Figure 2).

### Post-implementation assessment

Post implementation follow-up survey showed that 95.6% of focal points and 62.7% of clinicians knew the AFP case definition. The finding represents a 15.6% and 13.8% improvement for AFP focal points and clinicians respectively. Approximately one third (30.5%) of clinicians had knowledge of active case search which showed a substantial increase when compared with the baseline survey. In contrast to the baseline survey, an increase of 10.3% of clinicians at the post-intervention survey knew who to contact after a child with suspected AFP was diagnosed (Table 2). Table 3 shows pre- and post-study KAP scores amongst focal points. Clinicians showed an improvement in practice which was statistically significant (p<0.05). However, no statistically significant difference was found in knowledge and attitude of clinicians as well as attitude and practice of AFP focal points in pre and post intervention.

Three quarters (75%) of focal points in the follow-up assessment indicated they reported zero cases of AFP through the AFP SMS reminder system. Fifty-five percent of focal points indicated “I don’t receive SMS credit” as a reason for not reporting through the SMS system. In the follow-up assessment, 45% and 37% of clinicians indicated that regular training and stipend respectively were sufficient motivation for AFP SMS reporting. Approximately half (46%) of focal points indicated that stipends were sufficient motivation for reporting (Table 4).

## Discussion

The SMS AFP surveillance project with baseline survey, SMS reporting, and on-site training contributed to polio surveillance in multiple ways. First of all, the project contributed to identifying and addressing the knowledge gaps amongst health care workers. The baseline survey found that a significant proportion of health care workers lacked the important knowledge required for adequate AFP surveillance. A significant proportion of health care workers lacked the important knowledge required for accurate identification, investigation and reporting of AFP, which is consistent with other studies^8^,^9^.

At the baseline, most of these health care workers had never received formal training on AFP surveillance probably due to the significant attrition of healthcare workers and infrequent training given by the polio programme. The improvement in the follow-up survey indicated that the study strengthened AFP awareness and training to conventional AFP surveillance sites. Lastly, the initial survey of health facilities identified that 112 out of 127 focal sites in the state were operational, and a list of AFP reporting facility and focal points was updated accordingly.

More than 50% of focal points responded to SMS reminders, demonstrating the feasibility of this approach. Some health care workers responded to the SMS message in varied frequencies for lack of airtime credit. Although the use of SMS has been proven to yield positive results in reporting rate and surveillance^7^,^10^–^12^, the incentives may introduce the risk of falsification of results and could be more effective if a toll-free SMS system is deployed.

The study showed no significant increase in non-polio AFP reported during the study. This is probably because the AFP reporting rate is already high in Sokoto^4^ and there were not many missed AFP cases to be reported. The result is supported by the fact that no missed AFP cases were found during the active case search during the post assessment visits. A similar study in Papua New Guinea demonstrated that the use of SMS reporting increased the detection of measles and AFP cases, suggesting that this approach is useful in the areas with low reporting^13^,^14^. Apart from a high baseline reporting rate, the health care worker strike in Sokoto state which lasted for four out of the nine months of this study could have affected the reporting rate^15^.

**Figure 3:**
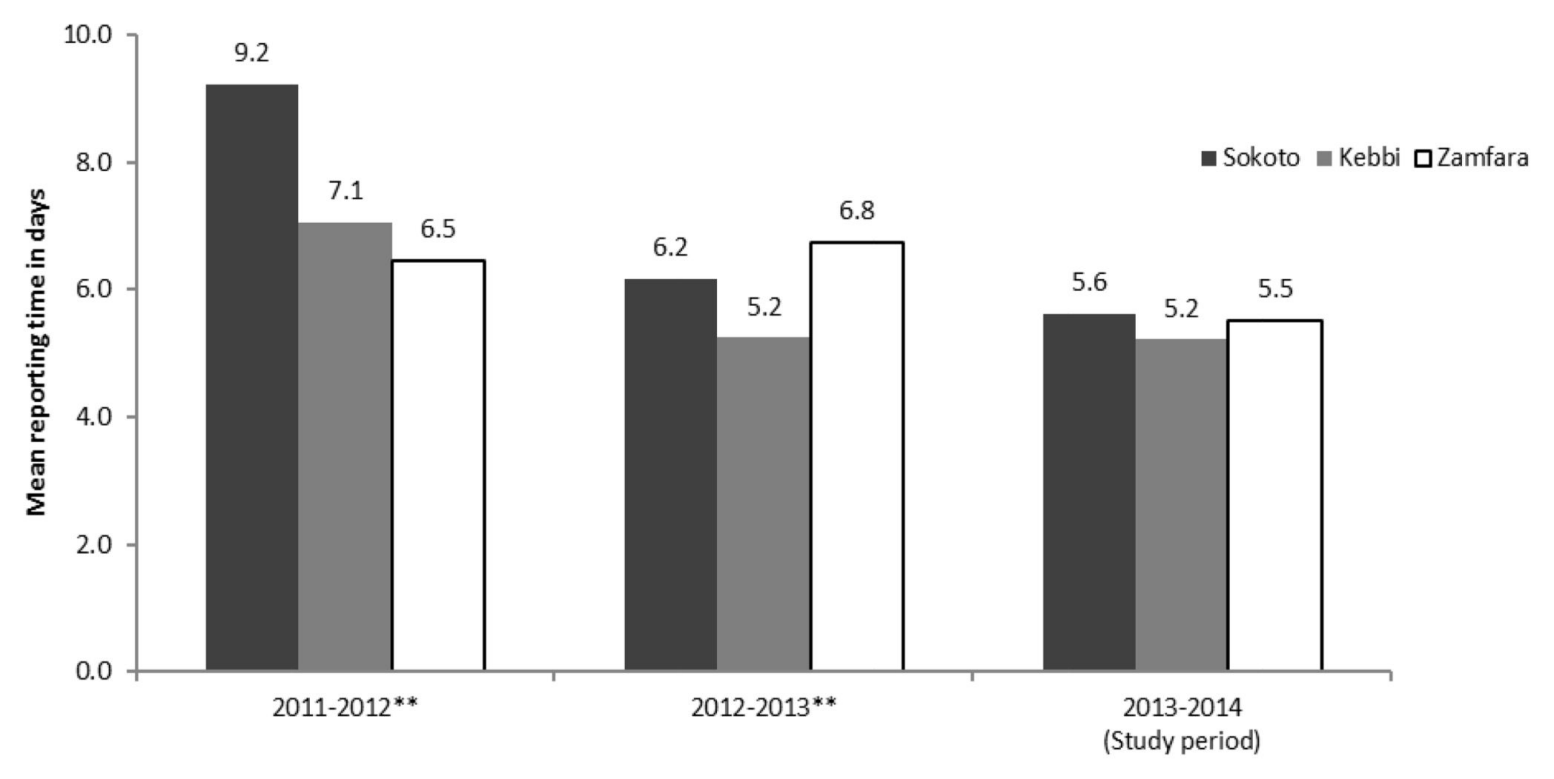
Trend in average days from AFP onset to reporting in neighbouring states

The number of days between the date of onset and date of reporting showed a slight decrease in time. However, this trend might be due to improvement in the reporting system over the years and may not solely be attributed to the SMS intervention. Indeed, this is likely the case as a similar trend was observed in Kebbi State, a nearby state without any SMS or training intervention (Figure 3).

As next steps, the study should be expanded to cover non-conventional health care providers and community informants, which is not part of the official surveillance system, and to areas with low AFP reporting rate, where there is more opportunity to identify unreported cases. In particular, the inclusion of these non-conventional informants amongst underserved and hard to reach populations would provide a unique opportunity to overcome access challenges^16^.

## Conclusion

The use SMS for AFP reporting improves reporting rate and timeliness. This innovative approach performs as well as the regular method. It remains a low-cost and efficient approach for surveillance activities. This approach would be a useful tool to improve AFP surveillance in difficult to reach, access compromised or countries/places without sufficient surveillance staff.

## Figures and Tables

**Table 1:** List of Knowledge, Attitude and Practice (KAP) questions

Category	Questions
Knowledge	Can you define the “AFP case definition”?
	Do you know what an active case search is?
	Have you been formally trained on AFP surveillance?
	Do you know who to contact in case you see a child with AFP
	How would you store and transport stool specimen?
Attitude	Do you believe polio is a serious public health issue?
	Is reporting AFP a priority in your work?
Practice	Do you have an AFP poster displayed in your health facility?
	Do the posters include a contact number for reporting AFP cases?

**Table 2:** Proportion of correctly answered questions by category

Categories	Questions	Clinicians		Focal Points	
		Before, n=223	After, n=84	Before, n=106	After, n=91
		n (%)	n (%)	n (%)	n (%)
Knowledge	Can you define the “AFP case definition”?	109 (48.9)	53 (62.7)	85 (80)	87 (95.6)
	Do you know what an active case search is?	44 (19.7)	26 (30.5)	-	-
	Have you been formally trained on AFP surveillance?	106 (47.5)	33 (39.3)	89 (83.9)	83 (91.2)
	Do you know who to contact in case you see a child with AFP	184 (82.5)	78 (92.8)	-	-
	How would you store and transport stool specimen?	-	-	89 (83.8)	80 (87.9)
Attitude	Do you believe polio is a serious public health issue?	219 (98.2)	84 (100)	105 (99)	91 (100)
	Is reporting AFP a priority in your work?	222 (99.5)	81 (96.4)	105 (99)	87 (95.6)
Practice	Do you have an AFP poster displayed in your health facility?	195 (87.4)	78 (92.9)	98 (92.5)	74 (81.3)
	Do the posters include a contact number for reporting AFP cases?	167 (74.9)	77 (91.7)	93 (87.7)	76 (83.5)

**Table 3:** Changes in Knowledge, attitude and practice scores, before and after the study (Mean score in each variable, scale of 0-8)

Variables	Clinicians				Surveillance focal points		
	Before, n=223	After, n=84	p value		Before, n=106	After, n=91	p value
	mean(SD)	mean(SD)			mean(SD)	mean(SD)	
Knowledge	1.99 (1.09)	2.07(1.08)	0.54		2.52(0.75)	2.74(0.63)	0.03
Attitude	1.98(0.18)	1.95(0.21)	0.34		1.98(0.19)	1.96(0.21)	0.38
Practices	1.62(0.69)	1.85(0.48)	<0.01		1.80(0.49)	1.65(0.67)	0.07

**Table 4:** Motivation for continued reporting by HCW during follow assessment

Motivation to report	Clinician, n= 177*	Focal point, n=107*
	n (%)	n (%)
Stipends	**66(37.3)**	**49(45.8)**
Awards & recognition	6(3.4)	3(2.8)
Regular meetings with program managers	9(5.1)	2(1.9)
Regular training & re-training	**79(44.6)**	**25(23.4)**
Supervision	13(7.3)	12(11.2)
Others	10(5.6)	16(15.0)

* Includes HCW who did not participate in the baseline assessment
